# No evidence of a genetic causal relationship between ankylosing spondylitis and iron homeostasis: A two-sample Mendelian randomization study

**DOI:** 10.3389/fnut.2023.1047640

**Published:** 2023-03-23

**Authors:** Mingyi Yang, Hui Yu, Ke Xu, Jiale Xie, Haishi Zheng, Ruoyang Feng, Jiachen Wang, Peng Xu

**Affiliations:** Department of Joint Surgery, HongHui Hospital, Xi’an Jiaotong University, Xi’an, Shaanxi, China

**Keywords:** ankylosing spondylitis, iron homeostasis, causal, Mendelian randomization, single nucleotide polymorphisms

## Abstract

**Background:**

Ankylosing spondylitis (AS) is an immune-mediated chronic inflammatory disease that leads to bone hyperplasia and spinal ankylosis. Iron homeostasis plays a very important role in the inflammatory response and is closely related to the pathogenesis of AS. This study aimed to use large-scale genome-wide association study (GWAS) summary data to study the genetic causal relationship between AS and iron homeostasis using Mendelian randomization (MR).

**Methods:**

Genome-wide association study summary data of AS and iron homeostasis-related indicators were obtained from the FinnGen consortium and the DeCODE genetics database, respectively. We used four iron homeostasis-related indicators: ferritin, serum iron, total iron binding capacity (TIBC), and transferrin saturation (TSAT) for two-sample MR analyses to test for genetic causal association with AS using the “TwoSampleMR” package of the R software (version 4.1.2). The random-effects inverse variance weighted (IVW) method was the main analysis method used for MR. We examined the MR analysis results for heterogeneity, horizontal pleiotropy, and possible outliers. In addition, we confirmed the robustness of the MR analysis by testing whether the results were affected by a single SNP and whether they followed a normal distribution.

**Results:**

The random-effects IVW results showed that ferritin [*p* = 0.225, OR 95% confidence interval (CI) = 0.836 (0.627–1.116)], serum iron [*p* = 0.714, OR 95% CI = 0.948 (0.714–1.260)], TIBC [*p* = 0.380, OR 95% CI = 0.917 (0.755–1.113)], and TSAT [*p* = 0.674, OR 95% CI = 0.942 (0.713–1.244)] have no genetic causal relationship with AS. We detected no heterogeneity，horizontal pleiotropy and possible outliers in our MR analysis (*p* > 0.05). In addition, our MR analysis results were not affected by a single SNP, and were normally distributed.

**Conclusion:**

Our study did not detect a genetic causal relationship between AS and iron homeostasis. Nonetheless, this does not rule out a relationship between the two at other mechanistic levels.

## 1. Introduction

Ankylosing spondylitis (AS) is a chronic inflammatory disease of unknown etiology. The disease is mainly characterized by the involvement of the spine and sacroiliac joints, eventually causing bone remodeling and spinal stiffness (1). Several factors are associated with AS risk, including genetic susceptibility (e.g., the human leukocyte antigen [*HLA]-B27* gene, which has the closest genetic relationship with AS), gender, autoimmunity, autoinflammation, bacterial infection, and biomechanics (2). Epidemiological studies have shown that the incidence of AS in the general population is approximately 0.09–0.3%, with geographic and ethnic variations (3). The main drugs used to treat AS are nonsteroidal anti-inflammatory drugs (NSAIDs), disease modifying antirheumatic drugs (DMARDs), glucocorticoids (GCS), tumor necrosis factor (TNF) inhibitors, and interleukin-17 (IL-17) inhibitors (4, 5). NSAIDs remain the first-line treatment, despite their gastrointestinal and cardiovascular side effects. As the disease progresses, surgery may be required to relieve symptoms (5), placing a physical and financial strain on the patient. Therefore, we examined the relationship between AS and other risk factors to clarify its etiological mechanism and provide new insights for the diagnosis and treatment of AS patients.

The common element iron is essential for several biochemical and physiological functions in the human body, and its metabolism needs to be carefully controlled (6). Iron homeostasis is regulated by a series of serum proteins, cellular receptors, hormones, and various other factors (7). Some of the most important iron-dependent biochemical pathways include mitochondrial respiration, metabolic processes, hormone synthesis, and deoxyribonucleic acid (DNA) synthesis (8). Iron homeostasis has been associated with various diseases, immune regulation, and the inflammatory response (6, 9). Immune activation in response to microbes, auto-antigens, or tumor antigens stimulates release of several pro-inflammatory cytokines by immune cells, which then cause disturbances in iron homeostasis (10). Interferon gamma (IFN-γ), lipopolysaccharide (LPS) and tumor necrosis factor alpha (TNF-α) upregulate the expression of divalent metal transporter 1 (DMT1) and downregulate the expression of ferroportin 1 (FPN1), resulting in subsequent increased iron uptake and reduced iron release from pro-inflammatory macrophages of the reticuloendothelial system (11). FPN1 is also expressed in duodenal enterocytes, therefore its downregulation under inflammatory conditions also limits intestinal iron absorption (11). Simultaneously, IL-1β, IL-6 and anti-inflammatory cytokines such as IL-4, IL-13, and IL-10 increase uptake of transferrin-bound iron (TFBI) and upregulate synthesis of the iron storage protein ferritin by various mechanisms (8). Inflammation also upregulates the expression of the iron-regulatory protein hepcidin (8). It can be seen that iron homeostasis is closely related to the inflammatory mechanism. AS an autoinflammatory disease, iron homeostasis must also be involved in the pathogenesis of AS.

Patients with AS may exhibit abnormal iron accumulation. A study using nuclear microprobe technology measured the cellular storage of iron in granulocytes and platelets of 29 patients with AS, and found high levels of iron in granulocytes and platelets (12). Another two studies of blood cells in patients with AS also found that iron accumulation was significant in granulocytes and platelets in patients with AS (13, 14). A case–control study of 30 patients with AS and 147 age - and sex-matched healthy controls found that iron intake was higher in patients with AS and that iron accumulation was significant in patients with AS (15). Iron overload in polymorphonuclear cells (PMNs) and platelets in AS patients, iron content in PMNs and platelets is negatively correlated with serum transferrin receptor (TfR) level, while iron content in PMNs is positively correlated with inflammation level (12). A serum proteomics and metabolomics study showed that serum TfR levels were significantly down-regulated in patients with AS (16). In addition, the incidence of iron deficiency-anemia in patients with AS is very high (17). We have recognized increased oxidative stress and lipid peroxidation in the pathogenesis of AS (18). Studies have analyzed the total antioxidant status (TAS), total oxidative status (TOS) and oxidative stress index (OSI) in plasma of AS patients, and the results suggest that the oxidation and antioxidant system disorders exist in AS patients (19). Blood markers of oxidative stress including superoxide dismutase (SOD), catalase (CAT), and nitric oxide (NO) were elevated in patients with AS combined with metabolic syndrome (MetS), but glutathione peroxidase (GPX) and TOS levels were not significantly changed (20). However, the serum GPX level was decreased in AS model mice (21), while reactive oxygen species (ROS) and malonaldehyde (MDA) levels in perivertebral connective tissue were significantly increased (22). In addition, increased levels of oxidized protein products (AOPPs) and ROS in serum of AS patients have been found to induce mesenchymal stem cells (MSCs) mitochondrial dysfunction in vitro (23). These evidence of oxidative stress and mitochondrial dysfunction in AS patients inform the existence of ferroptosis in AS (18). These studies indicate that iron homeostasis is involved in the pathogenesis of AS, and the study of the correlation between the two is of potential value for the study of the mechanism and prevention of AS. However, a direct causal relationship between the two has not been established. For this reason, we sought to analyze this issue in depth on a genetic level.

Mendelian randomization (MR) uses the genetic characteristics of random assignment of parental genes to offspring to study the causal relationship between exposure factors and outcomes (24). MR uses single nucleotide polymorphisms (SNPs) related to genetic variation as instrumental variables (IVs) of exposure factors. In this way, the analysis avoids adverse effects caused by environmental factors, reverse causality, and other factors (25). A recent MR study of iron levels and rheumatoid arthritis found an inverse relationship between the two (26). Nonetheless, MR has not been used to study the correlation between iron homeostasis and AS. In our study, ferritin, serum iron, total iron binding capacity (TIBC), and transferrin saturation (TSAT) were used as serum indicators to evaluate iron homeostasis. Two-sample MR analyses of each of the four indicators with AS were performed to evaluate the causal relationship between iron homeostasis and AS. In addition, strengthening the reporting of observational studies in epidemiology using Mendelian randomization (STROBE-MR) assists authors in reporting their MR research clearly and transparently. The STROBE-MR of this study can make readers more intuitively understand the whole process of the research (Table 1).

**Table 1:** STROBE-MR checklist of MR study.

**Item No**	**Section**	**Checklist item**
1	**Title**	No evidence of a genetic causal relationship between ankylosing spondylitis and iron homeostasis: a two-sample Mendelian randomization study
2	**Abstract**	AS is an immune-mediated chronic inflammatory disease that leads to bone hyperplasia and spinal ankylosis. Iron homeostasis plays a very important role in the inflammatory response and is closely related to the pathogenesis of AS. This study aimed to use GWAS summary data to study the genetic causal relationship between AS and iron homeostasis using MR. Our study did not detect a genetic causal relationship between AS and iron homeostasis. Nonetheless, this does not rule out a relationship between the two at other mechanistic levels
	**Introduction**	
3	Background	AS is a chronic inflammatory disease of unknown etiology. The disease is mainly characterized by the involvement of the spine and sacroiliac joints, eventually causing bone remodeling and spinal stiffness. Iron homeostasis is regulated by a series of serum proteins, cellular receptors, hormones, and various other factors. Iron homeostasis has been associated with various diseases, immune regulation, and the inflammatory response
4	Objectives	However, a direct causal relationship between the iron homeostasis and AS has not been established. For this reason, we sought to analyze this issue in depth on a genetic level
	**Methods**	
5	Study design	In this study, we selected SNPs from a GWAS dataset as IVs to investigate the causal relationship between AS and iron homeostasis related indicators (ferritin, serum iron, TIBC, TSAT)
6	Assumptions	
	(a)	All selected IVs were highly correlated with exposure (*P* < 5 × 10^−8^, F statistic >10)
	(b)	All selected IVs were independent of confounding factors between exposure and outcome
	(c)	All selected IVs affected outcomes by exposure only and not by other routes.
7	Data sources	
	(a)	GWAS summary data of iron homeostasis-related indicators were obtained from the DeCODE genetics database
	(b)	GWAS summary data of AS was obtained from the FinnGen consortium
8	MR analysis	
	(a)	Main method: random-effects IVW
	(b)	Supplementary methods: MR Egger, weighted median, simple mode, and weighted mode
	(c)	Verification methods: Maximum likelihood, penalized weighted median, IVW (fixed effects) and MR-RAPS
9	Sensitivity analysis	
	(a)	Heterogeneity: Cochran’s Q statistic for MR-IVW analyses and Rucker’s Q statistic for MR Egger analyses
	(b)	Horizontal pleiotropy: intercept test of MR Egger and global test of MR-PRESSO
	(c)	Outliers: distortion test of MR-PRESSO
	(d)	Leave one out: detect whether the causal relationship was influenced by a single SNP
	(e)	Normal distribution: MR-RAPS
10	Software	R software (version 4.1.2)
	**Results**	
11	Descriptive data	
	(a)	Ferritin and AS: 66 SNPs were used as IVs, including one palindromic SNP (rs2954029)
	(b)	Serum iron and AS: 36 SNPs were used as IVs, with no palindromic SNPs identified
	(c)	TIBC and AS: 46 SNPs were used as IVs, including two palindromic SNPs (rs11125072 and rs4854737)
	(d)	TSAT and AS: 46 SNPs were used as IVs, and there were no palindromic SNPs
12	Main results	
	(a)	Random-effects IVW: no genetic causal relationship
	(b)	MR Egger, Weighted median, Simple mode, and Weighted mode: no genetic causal relationship
	(c)	Maximum likelihood, Penalized weighted median, IVW (fixed effects) and MR-RAPS: no genetic causal relationship
13	Sensitivity analysis	
	(a)	No heterogeneity
	(b)	No horizontal pleiotropy
	(c)	No outliers
	(d)	The "leave one out" analysis indicated that the MR analysis results were not driven by a single SNP
	(e)	The MR analysis followed a normal distribution
	**Discussion**	
14	Key results	Our results showed that ferritin, serum iron, TIBC, and TSAT had no positive or negative causal relationships with AS with respect to genetic effects
15	Interpretation	
	(a)	The observed correlation between AS and iron homeostasis indicators may be related by inflammation-induced upregulation of hepcidin and AS-related anemia. Inflammation affects iron homeostasis indicators by increasing hepcidin and causes AS disease aggravation. Anemia may be an intermediary factor between AS and iron homeostasis indicators. Additionally, the anemia state caused by inflammation, complications, and treatment side effects of AS may causes changes in iron homeostasis indicators
	(b)	Considering this, inflammation or anemia may be simultaneously related to AS and the four iron homeostasis indicators in this study (ferritin, serum iron, TIBC, TSAT). AS-related anemia and inflammation-induced upregulation of hepcidin may account for the observed association between AS and the four iron homeostasis indicators. Nonetheless, this does not provide evidence of a direct genetic causal relationship between AS and iron homeostasis indicators. Furthermore, these indicators are susceptible to other diseases that cause inflammation and anemia
16	Limitations	
	(a)	The data were limited to patients of European ancestry, therefore the conclusions drawn may not be applicable to other populations
	(b)	Furthermore, there were relatively few iron homeostasis indicators in this study
	(c)	Finally, the exposure factors in this study were the effects of mutated genes, which may differ from the actual exposure in terms of time and dose
17	**Conclusion**	In conclusion, our results suggest that the four iron homeostasis indicators have no clear causal relationship with AS with respect to genetic factors. Moreover, there may be a correlation between AS and iron homeostasis indicators on other levels beyond genetics. Additional mechanistic links between the two remain unclear and should be further examined in future research
	**Other information**	
18	Conflict of Interest	There is no conflict of interest between all authors of this article
19	Funding	This work was financially supported by the National Natural Science Foundation of China (No. 82072432)
20	Data availability	The GWAS summary data used in this study were obtained from the FinnGen consortium (https://www.finngen.fi/en) and the DeCODE genetics database (https://www.decode.com/)

## 2. Materials and methods

### 2.1. Study design

In this study, we selected SNPs from a genome-wide association study (GWAS) dataset as IVs to investigate the causal relationship between exposure and outcome. This study satisfied three key assumptions of the two-sample MR design: (1) all selected IVs were highly correlated with exposure (*p* < 5 × 10^−8^, F statistic >10); (2) all selected IVs were independent of confounding factors between exposure and outcome; (3) all selected IVs affected outcomes by exposure only and not by other routes. All datasets used in this study are publicly available. Ethical permission and written informed consent was provided in the initial studies (27).

### 2.2. Genome-wide association study summary data for iron homeostasis-related indicators

Genome-wide association summary data of iron homeostasis-related indicators, including ferritin (*N* = 246,139), serum iron (*N* = 163,511), TIBC (*N* = 135,430), and TSAT (*N* = 131,471), were obtained from the DeCODE genetics database^1^ (28). All participants in the study were of European ancestry and provided informed consent. Ferritin measurement was based on the immunological agglutination principle and the reaction was enhanced by latex. Serum iron was measured using a colorimetric method (FerroZine) without deproteinization. TIBC was calculated by adding serum iron and unsaturated iron-binding capacity, which was also measured photometrically. TSAT was calculated by dividing serum iron by TIBC concentration. Quality control (QC) included exclusion of gender mismatches, low calling rates, duplicate samples, extreme heterozygosity, and non-European ancestry. We carried out high-resolution multiple imputations using a joint UK10K and the 1,000 Genomes Phase 3 (May 2013 release) reference panel and retained variants with a MAF ≥ 0.1% and/or INFO score ≥ 0.4 for analysis. The four iron homeostasis-related indicators (ferritin, serum iron, TIBC, and TSAT) were each rank-based inverse normal transformed to a standard normal distribution (separately for each sex) and adjusted for age using a generalized additive model. For each sequence variant, the mixed model implemented in the BOLT-LMM v2.3 software, using genotype as an additive covariate and transformed quantitative trait as the response, was used to test for association with quantitative traits (29). A logistic regression was used to test associations between variants and case–control phenotypes using the software developed at deCODE genetics (30). A linkage disequilibrium (LD) score regression was used to account for distribution inflation in the dataset due to cryptic correlations and population stratification (31). Further details of the data are available in a previously published study (28).

### 2.3. Genome-wide association summary data for ankylosing spondylitis

Genome-wide association summary data of AS were obtained from the FinnGen consortium.^2^ All participants were of European ancestry and informed consent was provided. The FinnGen research project is a public-private partnership that integrates disease endpoint genetic data provided by the Finnish Biobank and the Finnish Health Registry (32). The FinnGen research project aimed to identify genotype–phenotype correlations in the Finnish population. Additionally, it was designed to develop the potential of these resources to inform medical research and enrich drug discovery programs. We used the publicly available data release (freeze 7) which included 2,252 diagnosed AS patients and 227,338 controls of Finnish ancestry. All cases were defined using the M13 code in the International Classification of Diseases-Tenth Revision (ICD-10). These individuals were genotyped using Illumina (Illumina Inc., San Diego) and Affymetrix chip arrays (Thermo Fisher Scientific, Santa Clara, CA, United States), and 16,962,023 variants were analyzed in total. Detailed information on the participants, genotyping, imputation, and quality control can be found on the FinnGen website.^3^

### 2.4. Selection of instrumental variables

To ensure the accuracy and robustness of conclusions regarding the causal relationship between iron homeostasis-related indicators and AS, we employed a series of quality control steps to select valid IVs. First, we obtained SNPs associated (*p* < 5 × 10^−8^) with four iron homeostasis-related indicators (ferritin, serum iron, TIBC, and TSAT). Second, since strong LD among the selected SNPs may lead to biased results, the clumping process (r^2^ < 0.001, clumping distance = 10,000 kb) was carried out to eliminate the LD between the included IVs (32). Third, we excluded SNPs associated with outcome (AS; *p* < 5 × 10^−8^). Fourth, we applied the PhenoScanner database^4^ to assess whether the selected SNPs were associated with confounders (33). From previous literature and studies, we found that the main risk factors for AS are obesity, body mass index (BMI), smoking, and diabetes (34–38). Fifth, to satisfy the strong association with exposure, we selected SNPs with an F statistic >10 as IVs. F statistics were calculated using the formula: F = R^2^(N-K-1)/K(1-R^2^), and R^2^ was calculated using the formula: R^2^ = 2 × MAF×(1-MAF) Beta^2^. Sixth, palindromic SNPs with intermediate allele frequencies were excluded to guarantee that the impact of SNPs on exposure corresponds to the same allele as that providing the effect on outcome (39). Seventh, when SNPs were not available from the GWAS results, proxy SNPs were identified through the LD link online platform.^5^

### 2.5. Statistical analysis

The “TwoSampleMR” package of the R software (version 4.1.2) was used to perform two-sample MR analyses of iron homeostasis-related indicators and AS. For the MR analysis, we used the random-effects inverse variance weighted (IVW) as the main method, and the MR Egger, Weighted median, Simple mode, and Weighted modes as supplementary methods. The MR analysis results were therefore predominantly from random-effects IVW. In the absence of directional pleiotropy, the IVW method, which combines the Wald estimates for each IV through a meta-analytic approach, can provide relatively stable and accurate causal estimates (39). The MR Egger regression can detect pleiotropy through its intercept and provide estimates after correction for pleiotropy; however, it reduces statistical power. The Weighted median analysis is an important method to estimate causal effects if more than 50% of SNPs met the “no horizontal pleiotropy” assumption (40). The simple mode is a model-based estimation method that provides robustness for pleiotropy, although it is not as powerful as IVW (41). The Weighted mode is sensitive to the difficult bandwidth selection for mode estimation (42).

We used the Cochran’s Q statistic for MR-IVW analyses and Rucker’s Q statistic for MR Egger analyses to detect the heterogeneity of the effects of SNPs related to ferritin, serum iron, TIBC, and TSAT on AS outcomes, with *p* > 0.05 indicating no heterogeneity (43). We used the MR Egger method to assess the extent to which directional pleiotropy may affect risk estimates by intercept tests, with *p* > 0.05 indicating no horizontal pleiotropy (33). Since MR Egger may show lower accuracy in some cases, the MR pleiotropy residual sum and outlier (MR-PRESSO) method was also used to assess outlier SNPs and potential horizontal pleiotropy (33). The distortion test embedded in the MR-PRESSO analysis can detect outliers present in the MR analysis, and the global test embedded in the MR-PRESSO analysis can detect horizontal pleiotropy, with *p* > 0.05 indicating no horizontal pleiotropy (44). Importantly, any outliers in the distortion test were excluded and the causal estimates were reassessed. We also performed a “leave one out” analysis to investigate whether the causal relationship between exposure and outcome was influenced by a single SNP (45). Additionally, we applied the radial variants of the IVW and MR Egger models for improved visualization of the causal estimate (46). Moreover, the MR robust adjusted profile score (MR-RAPS) method not only assessed causality, but also validated the robustness of systemic and specific pleiotropy (46). A *p* value >0.05 indicated that the data conformed to the normal distribution and the evaluation results were strongly robust. Finally, we performed Maximum likelihood, Penalized weighted median, and IVW (fixed effects) to further identify the potential causal association between exposures and outcomes.

## 3. Results

### 3.1. Selection of instrumental variables

We identified 72 SNPs shared by ferritin and AS, and after excluding one SNP associated with AS and five SNPs associated with confounding factors, the remaining 66 SNPs were used as IVs (F statistic >10), including one palindromic SNP (rs2954029; Supplementary FigureSupplementary Table S1). We identified 39 SNPs shared by serum iron and AS, and after excluding two SNPs associated with AS and one SNP associated with confounding factors, the remaining 36 SNPs were used as IVs (F statistic >10), with no palindromic SNPs identified (Supplementary FigureSupplementary Table S2). We found 55 SNPs shared by TIBC and AS, excluded eight SNPs associated with AS and one SNP associated with confounding factors, and used the remaining 46 SNPs as IVs (F statistic >10), including two palindromic SNPs (rs11125072 and rs4854737; Supplementary FigureSupplementary Table S3). Finally, we identified 51 SNPs shared by TSAT and AS, excluded four SNPs associated with AS and one SNP associated with confounding factors; the remaining 46 SNPs were used as IVs (F statistic >10), and there were no palindromic SNPs (Supplementary FigureSupplementary Table S4).

### 3.2. Mendelian randomization analysis

**Figure 1:**
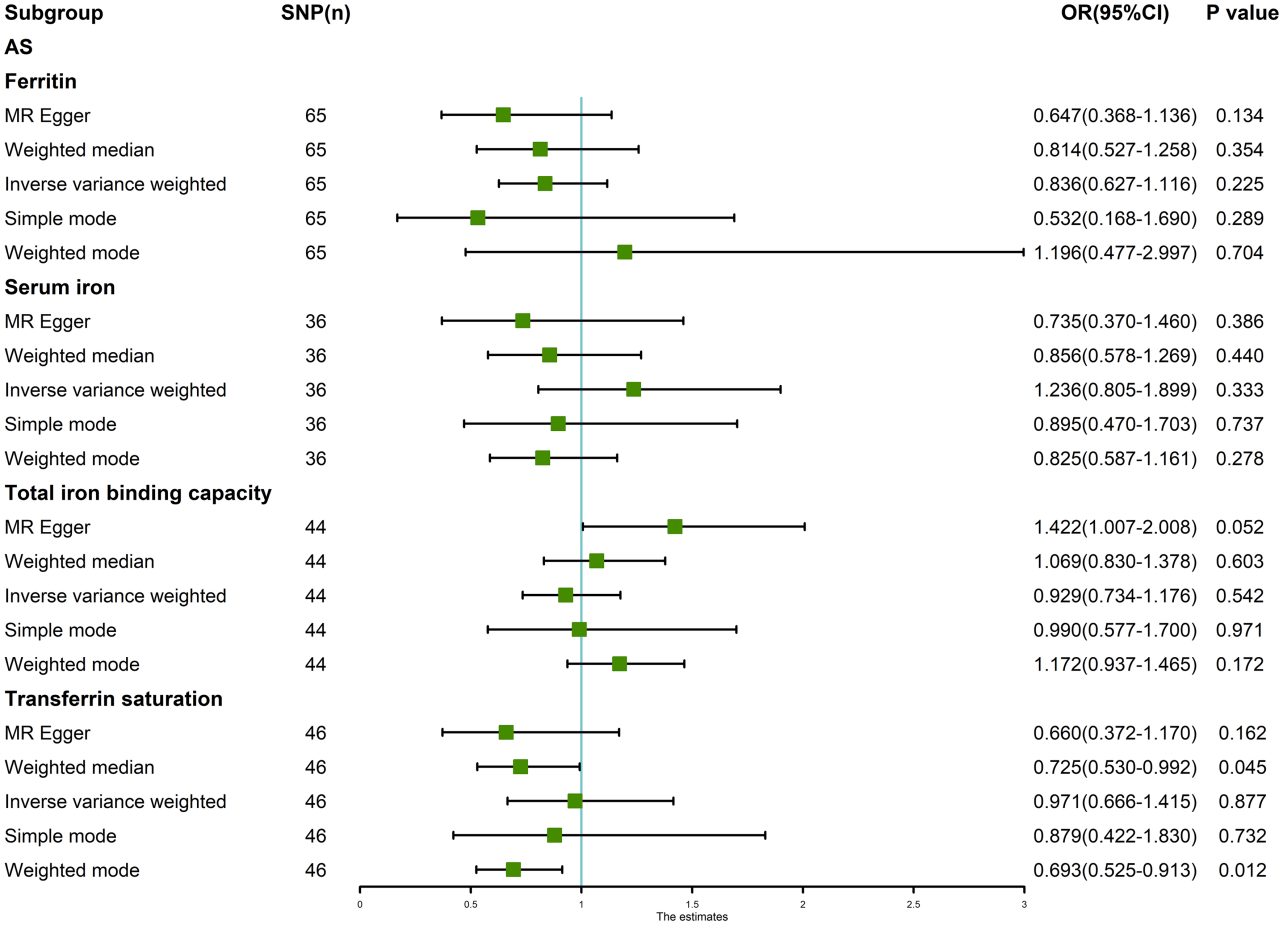
MR analysis results of the four exposures (ferritin, serum iron, total iron binding capacity, and transferrin saturation) and outcomes (ankylosing spondylitis). Five methods: random-effects IVW, MR Egger, weighted median, simple mode, and weighted mode.

**Figure 2:**
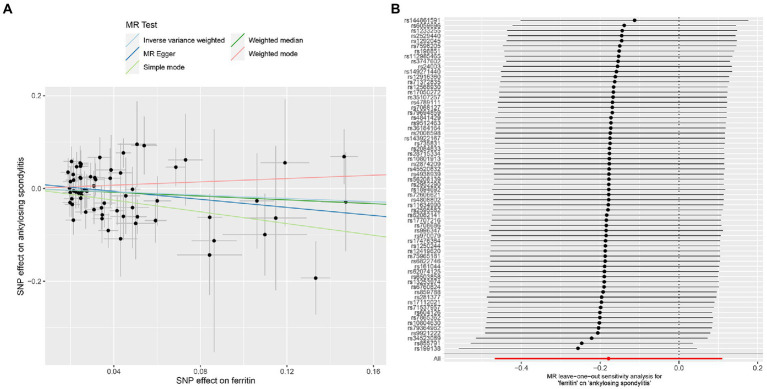
MR analysis of the ferritin and ankylosing spondylitis. **(A)** Scatter plot. **(B)** Leave one out analysis.

The random-effects IVW results showed that ferritin [*p* = 0.225, OR 95% confidence interval (CI) = 0.836 (0.627–1.116)], serum iron [*p* = 0.333, OR 95% CI = 1.236 (0.805–1.899)], TIBC [*p* = 0.542, OR 95% CI = 0.929 (0.734–1.176)], and TSAT [*p* = 0.877, OR 95% CI = 0.971 (0.666–1.415)] had no genetic causal relationship with AS (Figures 1, 2A; Supplementary FigureSupplementary Figure S1). The MR Egger, weighted median, simple mode, and weighted mode analysis showed that ferritin, serum iron, and TIBC had no genetic causal relationship with AS (*p* > 0.05). The MR Egger and Simple mode analysis showed that TSAT had no genetic causal relationship with AS (*p* > 0.05). However, the Weighted median and Weighted mode analysis showed that TSAT had a genetic causal relationship with AS (*p* < 0.05; Figure 1).

**Figure 3:**
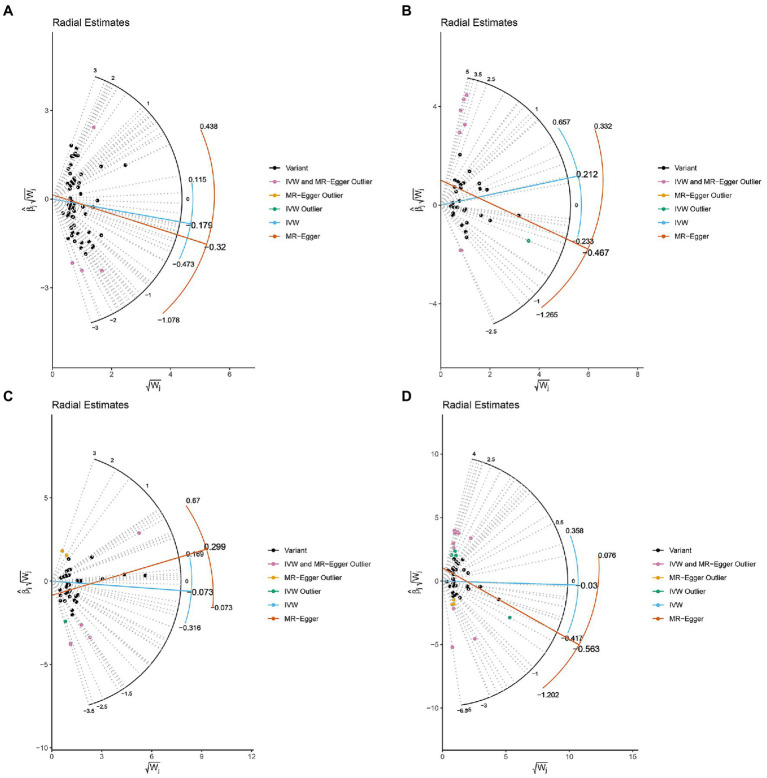
Radial plots to visualize individual outlier SNPs in the MR estimates. The radial curve displays the ratio estimate for each SNP. Black dots show valid SNPs. **(A)** MR radial plots of ferritin and ankylosing spondylitis. **(B)** MR radial plots of serum iron and ankylosing spondylitis. **(C)** MR radial plots of total iron binding capacity and ankylosing spondylitis. **(D)** MR radial plots of transferrin saturation and ankylosing spondylitis.

**Figure 4:**
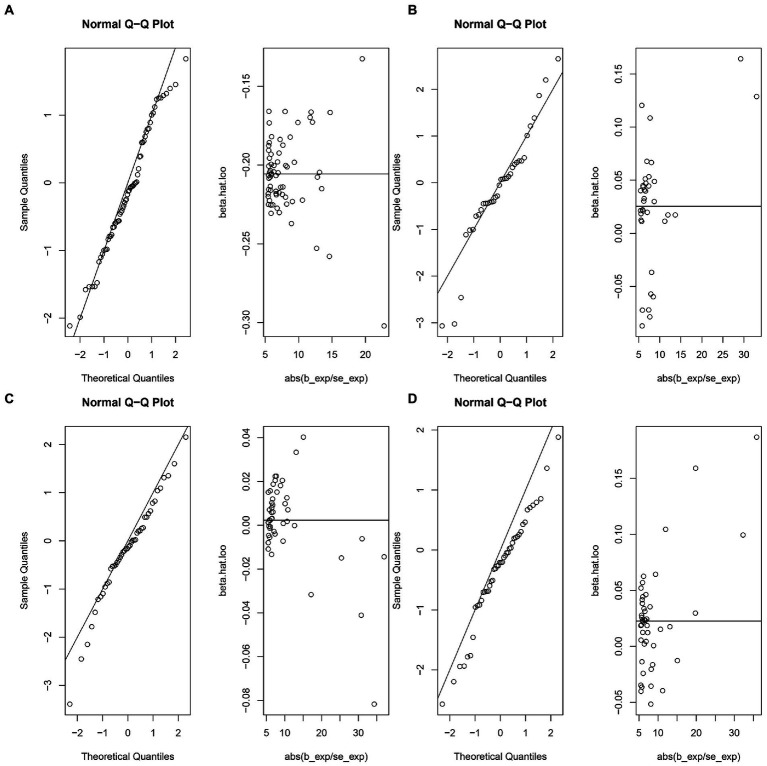
The normal distribution plots of MR analysis for exposure and outcome. **(A)** The normal distribution plots of MR analysis for ferritin and ankylosing spondylitis. **(B)** The normal distribution plots of MR analysis for serum iron and ankylosing spondylitis. **(C)** The normal distribution plots of MR analysis for total iron binding capacity and ankylosing spondylitis. **(D)** The normal distribution plots of MR analysis for transferrin saturation and ankylosing spondylitis.

The MR-IVW test showed that the MR analysis results of AS with ferritin (*p* = 0.098) displayed no heterogeneity, whereas the serum iron (*p* = 1.14E-07), TIBC (*p* = 3.99E-05), and TSAT (*p* = 1.35E-20) tested positive for heterogeneity. The MR Egger test also showed that the MR analysis results of ferritin (*p* = 0.101) displayed no heterogeneity, whereas the serum iron (*p* = 1.29E-06), TIBC (*p* = 0.002), and TSAT (*p* = 7.94E-19) displayed significant heterogeneity (Table 2). The results of the MR Egger test of horizontal pleiotropy showed that the MR analysis results of AS with ferritin (*p* = 0.302), serum iron (*p* = 0.071), and TSAT (*p* = 0.091) displayed no significant horizontal pleiotropy, whereas TIBC (*p* = 0.004) did display significant pleiotropy (Table 2). In contrast, the MR-PRESSO global test showed that the MR analysis results of AS with ferritin (*p* = 0.105) displayed no horizontal pleiotropy, whereas significant horizontal pleiotropy was detected for AS with serum iron (*p* < 0.001), TIBC (*p* < 0.001) and TSAT (*p* < 0.001; Table 2). The MR-PRESSO distortion test results showed that the number of outliers detected during the MR analysis with AS was 0, 4, 4, and 11 for ferritin, serum iron, TIBC, and TSAT, respectively (Table 2). The “leave one out” analysis indicated that the MR analysis results were not driven by a single SNP (Figure 2B; Supplementary FigureSupplementary Figure S2). In addition, the IVW and MR Egger radial MR method delineation showed that there were outliers in the MR analysis process of AS with ferritin, serum iron, TIBC, and TSAT (Figure 3). The MR-RAPS analysis showed that there was no genetic causal relationship with AS for ferritin (*p* = 0.194, OR = 0.814), serum iron (*p* = 0.893, OR = 1.016), TIBC (*p* = 0.984, OR = 1.002), or TSAT (*p* = 0.927, OR = 1.023; Table 2). In addition, the MR-RAPS analysis showed that the MR analyses of AS with ferritin (*p* = 0.355), TIBC (*p* = 0.319), or TSAT (*p* = 0.340) were normally distributed, whereas the MR analyses of AS with serum iron were not normally distributed (*p* = 0.040; Table 2; Figure 4).

**Table 2:** Sensitivity analysis of the MR analysis results of exposures and outcomes.

**Exposure**	**Outcome**	**Heterogeneity test**		**Pleiotropy test**	**MR-PRESSO**		**MR-RAPS**		
		**Cochran’s Q test (*P* value)**	**Rucker’s Q test (*P* value)**	**Egger intercept (*P* value)**	**Distortion test**	**Global test**	**MR analysis**		**Normal distribution**
		**IVW**	**MR Egger**	**MR Egger**	**Outliers**	***P* value**	**OR**	***P* value**	***P* value**
Ferritin	AS	0.098	0.101	0.302	NA	0.105	0.814	0.194	0.355
Serum iron	AS	1.14E-07	1.29E-06	0.071	4	<0.001	1.016	0.893	0.040
Total iron binding capacity	AS	3.99E-05	0.002	0.004	4	<0.001	1.002	0.984	0.319
Transferrin saturation	AS	1.35E-20	7.94E-19	0.091	11	<0.001	1.023	0.927	0.340

**Figure 5:**
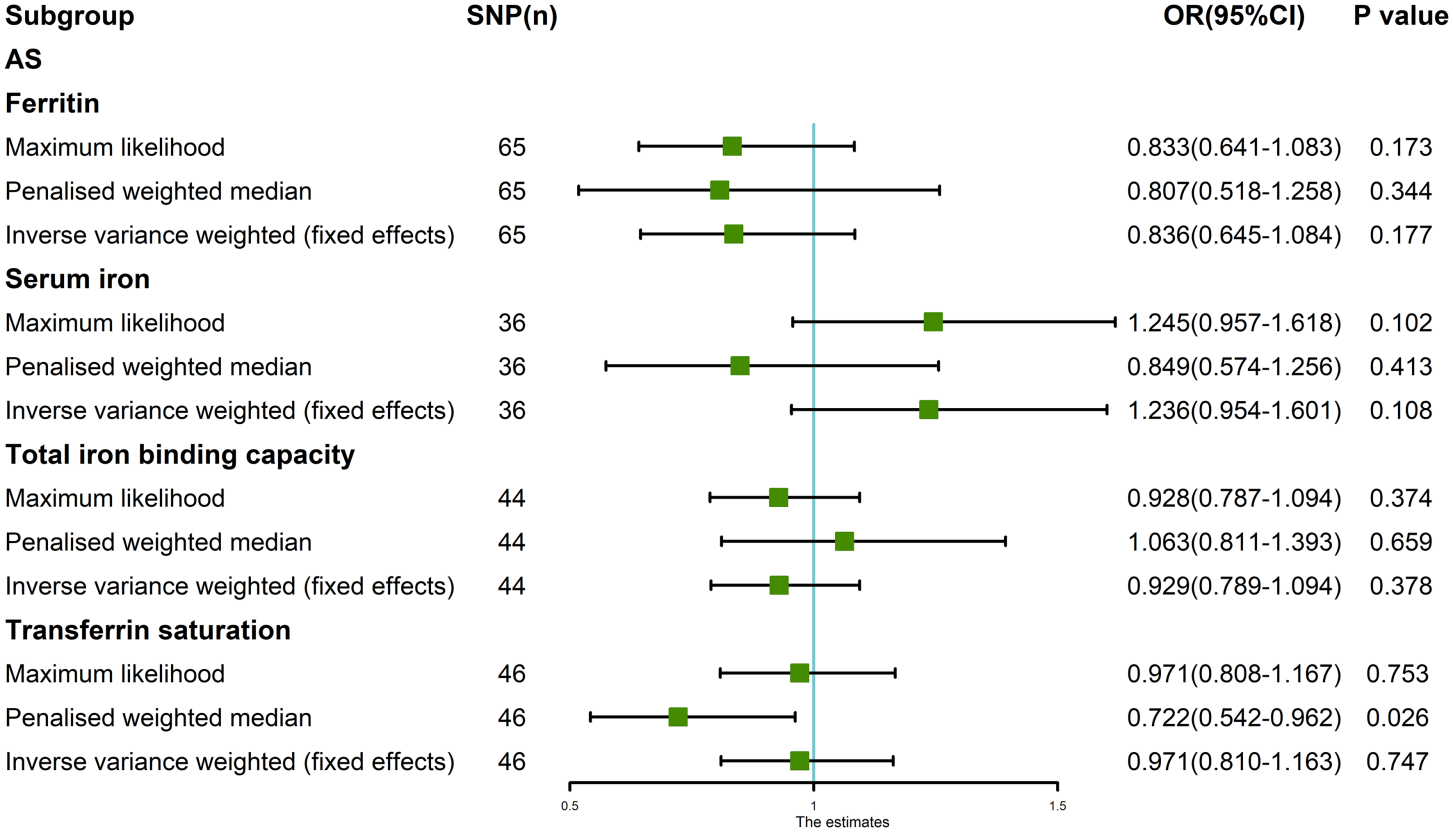
The MR analysis between four exposures (ferritin, serum iron, total iron binding capacity, transferrin saturation) and outcomes (ankylosing spondylitis). Three methods: Maximum likelihood, Penalized weighted median, and IVW (fixed effects).

The Maximum likelihood, Penalized weighted median, and IVW (fixed effects) results showed that ferritin, serum iron, and TIBC had no genetic causal relationship with AS (*p* > 0.05). The Maximum likelihood and IVW (fixed effects) results showed that TSAT had no genetic causal relationship with AS (*p* > 0.05), but the Penalized weighted median results showed that TSAT had genetic causal relationship with AS (*p* < 0.05; Figure 5).

### 3.3. Mendelian randomization analysis after removing outliers

We wondered whether the heterogeneity and horizontal pleiotropy detected in the MR analysis of serum iron, TIBC, and TSAT with AS had been affected by the outliers that were detected by MR-PRESSO. Therefore, we performed MR analysis again after removing the outliers detected by MR-PRESSO, to evaluate the genetic association of serum iron, TIBC, and TSAT with AS.

**Figure 6:**
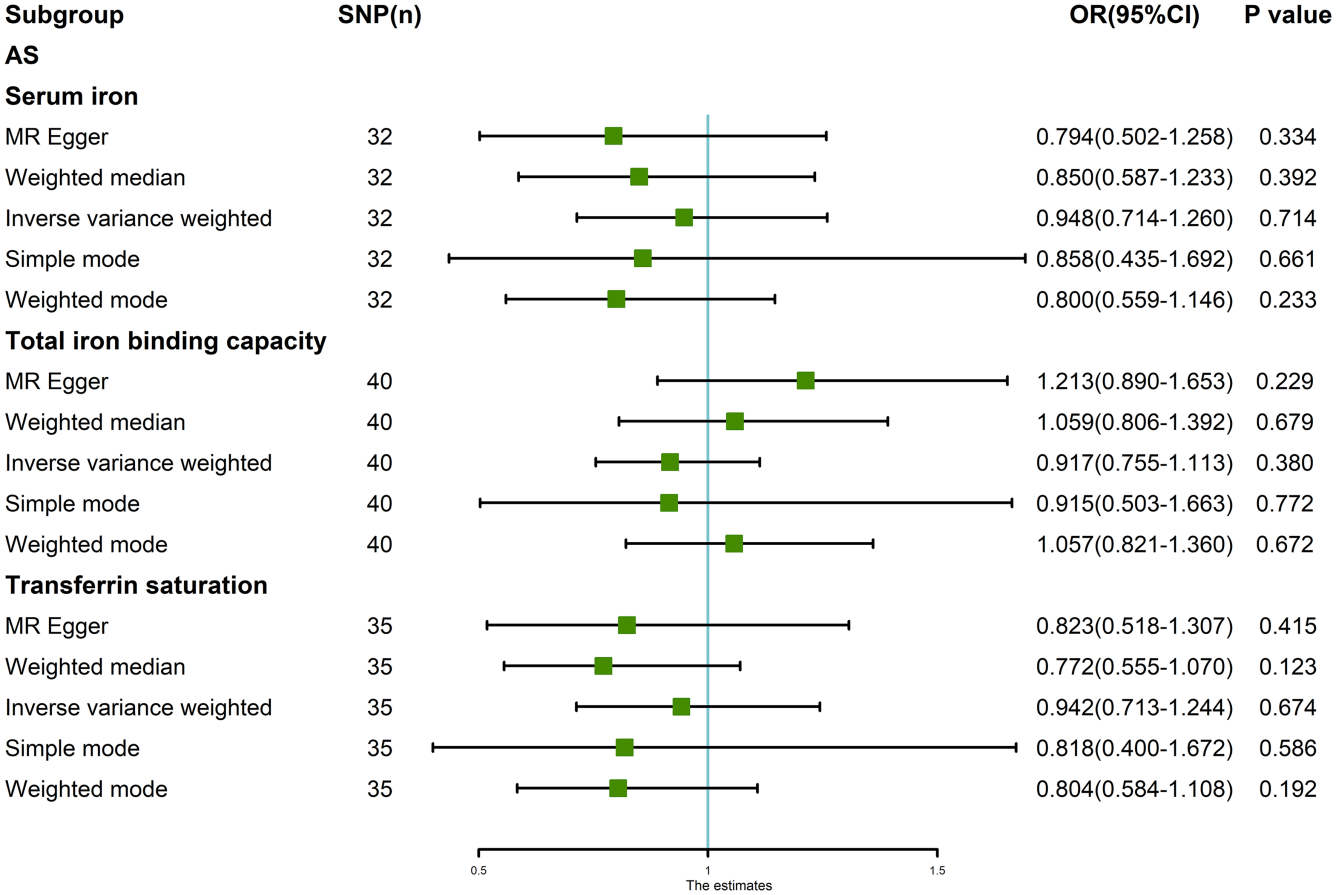
MR analysis results of the three exposures (serum iron, total iron binding capacity, transferrin saturation) and outcomes (ankylosing spondylitis) after exclusion of outlier SNPs detected by MR-PRESSO.

**Figure 7:**
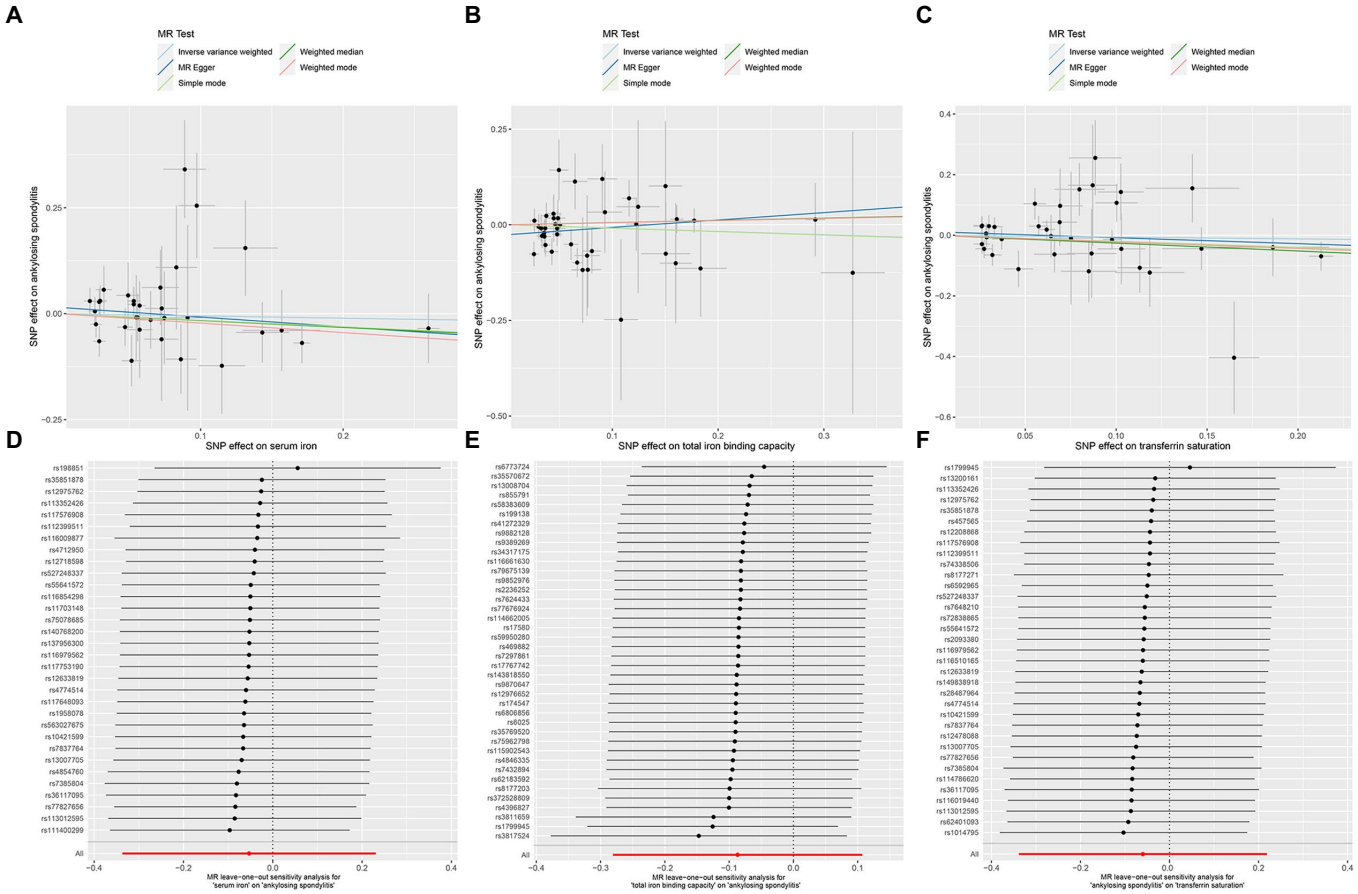
MR analysis of the three exposures (serum iron, total iron binding capacity, transferrin saturation) and outcomes (ankylosing spondylitis) after exclusion of outlier SNPs. **(A)** Scatter plot of serum iron and ankylosing spondylitis. **(B)** Scatter plot of total iron binding capacity and ankylosing spondylitis. **(C)** Scatter plot of transferrin saturation and ankylosing spondylitis. **(D)** Leave one out analysis of serum iron and ankylosing spondylitis. **(E)** Leave one out analysis of total iron binding capacity and ankylosing spondylitis. **(F)** Leave one out analysis of transferrin saturation and ankylosing spondylitis.

After removing outliers, the random-effects IVW results showed that serum iron [*p* = 0.714, OR 95% CI = 0.948 (0.714–1.260)], TIBC [*p* = 0.380, OR 95% CI = 0.917 (0.755–1.113)], and TSAT [*p* = 0.674, OR 95% CI = 0.942 (0.713–1.244)] had no genetic causal relationship with AS (Figures 6, 7A–C). The MR analysis results of the MR Egger, weighted median, simple mode, and weighted mode methods were consistent with those of the random-effects IVW method (*p* > 0.05; Figure 6).

After removing outliers, the MR-IVW heterogeneity test showed that there was no heterogeneity in the MR analysis of AS with serum iron (*p* = 0.281), TIBC (*p* = 0.357), or TSAT (*p* = 0.061). The MR Egger heterogeneity test also showed that there was no heterogeneity in the MR analysis of AS with serum iron (*p* = 0.280), TIBC (*p* = 0.531), or TSAT (*p* = 0.056; Table 3). The results of the MR Egger test of horizontal pleiotropy showed that there was no horizontal pleiotropy in the MR analysis results of AS with serum iron (*p* = 0.344), TIBC (*p* = 0.132), or TSAT (*p* = 0.477; Table 3). The “leave one out” analysis indicated that the MR analysis results were not driven by a single SNP (Figures 7D–F). The MR-PRESSO analysis detected no outliers in the MR results, and showed that there was no horizontal pleiotropy in the MR analysis results of AS with serum iron (*p* = 0.314), TIBC (*p* = 0.345), or TSAT (*p* = 0.086; Table 3). The MR-RAPS analysis showed that serum iron (*p* = 0.566, OR = 0.922), TIBC (*p* = 0.961, OR = 0.995), and TSAT (*p* = 0.576, OR = 0.916) had no genetic causal relationship with AS (Table 3), and that all three analyses followed a normal distribution (*p* = 0.079, 0.739, 0.922 for serum iron, TIBC, and TSAT, respectively; Table 3, Supplementary FigureSupplementary Figure S3).

**Table 3:** Sensitivity analysis of the MR analysis results of exposures and outcomes after exclusion of outlier SNPs detected by MR-PRESSO.

**Exposure**	**Outcome**	**Heterogeneity test**		**Pleiotropy test**	**MR-PRESSO**		**MR-RAPS**		
		**Cochran’s Q test (*P* value)**	**Rucker’s Q test (*P* value)**	**Egger intercept (*P* value)**	**Distortion test**	**Global test**	**MR analysis**		**Normal distribution**
		IVW	MR Egger	MR Egger	Outliers	*P* value	OR	*P* value	*P* value
Serum iron	AS	0.281	0.280	0.344	NA	0.314	0.922	0.566	0.079
Total iron binding capacity	AS	0.357	0.531	0.132	NA	0.345	0.995	0.961	0.739
Transferrin saturation	AS	0.061	0.056	0.477	NA	0.086	0.916	0.576	0.922

**Figure 8:**
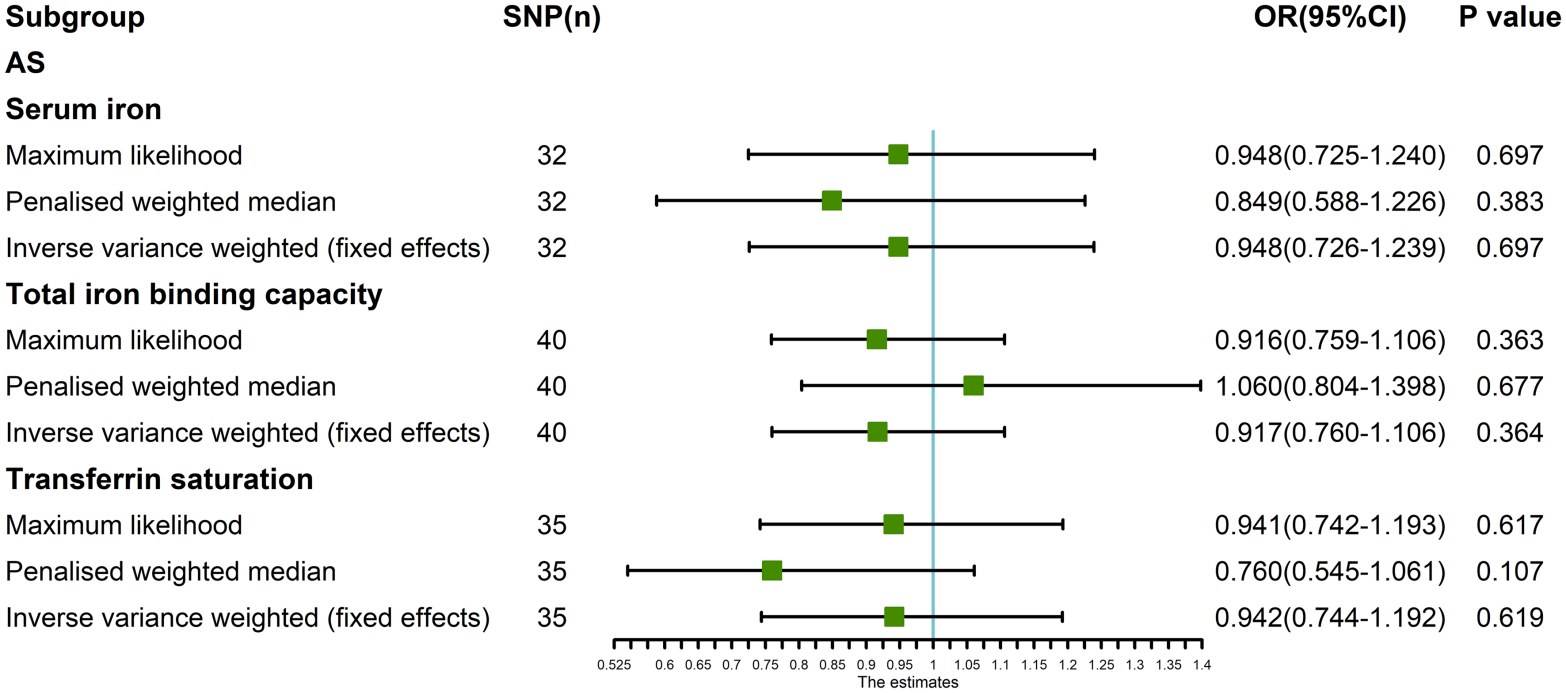
The MR analysis between three exposures (serum iron, total iron binding capacity, transferrin saturation) and outcomes (ankylosing spondylitis) after exclusion of outlier SNPs detected by MR-PRESSO. Three methods: Maximum likelihood, penalized weighted median, and IVW (fixed effects).

In addition, the Maximum likelihood, Penalized weighted median, and IVW (fixed effects) results showed that serum iron, TIBC, and TSAT had no genetic causal relationship with AS (*p* > 0.05; Figure 8).

## 4. Discussion

To our knowledge, this study is the first to analyze the causal relationship between iron homeostasis and AS based on two-sample MR. Our results showed that ferritin, serum iron, TIBC, and TSAT had no positive or negative causal relationships with AS with respect to genetic effects. This study relied on a large sample size from the GWAS summary data and on a two-sample MR analysis to avoid sample overlap. Furthermore, we minimized confounding factors and eliminated heterogeneity and horizontal pleiotropy in the MR analysis. Therefore, our results were robust and satisfied the three assumptions of MR.

Only two studies have investigated the relationship between these four metrics and AS, and both differed from the present study. The first was a prospective study that reported significantly higher serum hepcidin levels in 40 AS patients than in healthy controls. Serum iron, TIBC, and TSAT levels were significantly lower in AS patients than in healthy controls, while the levels of ferritin were not significantly different (47). A second, more recent study on 76 AS patients (divided into anemia, non-anemia, and control groups) demonstrated that in the anemia group, the levels of ferritin, serum iron, and TSAT increased with increasing hepcidin levels (48). These observational findings differed from our results, possibly because these studies were affected by inflammation-induced upregulation of hepcidin and AS-related anemia.

Inflammation in AS patients can induce increased hepcidin levels through a variety of inflammatory factors (49). Hepcidin binds directly to FPN1, the only iron export protein on the cell surface, which can change its conformation or induce its degradation by endocytosis, thereby reducing its ability to export iron from cells (7, 50). This leads to iron accumulation in cells, and the resulting serum iron deficiency causes changes in serum iron homeostasis indicators (51, 52). FPN1 is mainly expressed on the surface of intestinal epithelial cells and macrophages, but is also expressed on the surface of osteoblasts. The iron content in osteoblasts therefore also increases with increasing levels of hepcidin (53). Therefore, persistent high hepcidin levels due to chronic inflammation in patients with AS may reduce FPN1 in osteoblasts, resulting in increased iron content. A continuously high iron content in osteoblasts can result in increased oxidative stress due to excessive reactive oxygen species (ROS) production *via* the Fenton reaction, leading to osteoblast damage and dysfunction (54, 55). This process affects bone metabolism in AS patients. A retrospective study found that patients with AS commonly have low bone mineral density (BMD), which may aggravate the symptoms of AS (56). Therefore, the observed correlation between AS and iron homeostasis indicators may be mediated by increased hepcidin levels during inflammation, which affects iron homeostasis indicators leading to aggravation of AS disease.

Anemia in AS patients is predominantly one of two types; anemia of chronic disease (ACD) and iron deficiency anemia (IDA) (17, 57). ACD is caused by high levels of hepcidin induced by inflammation, which reduces cells’ ability to release iron, causing relative iron deficiency in serum and indirectly inhibiting erythropoiesis (58). The main indicators of iron homeostasis in ACD patients are high serum hepcidin, high or normal ferritin (caused by an inflammatory response), low serum iron, and TSAT (10, 58). IDA is an absolute iron deficiency caused by chronic blood loss or malabsorption of iron in the digestive tract (59). Indicators of iron homeostasis in patients are low serum hepcidin (which may favor iron release from cells), low ferritin, serum iron, and TSAT, and high TIBC (48, 60). ACD may occur in patients with AS who are in a state of long-term inflammation (57). Inflammatory bowel disease (IBD) and/or the gastrointestinal side effects of NSAIDs in AS patients with ACD can trigger iron malabsorption, gastrointestinal bleeding, and IDA (10). This decreases the level of hepcidin compared to that in ACD or IDA alone, whereas ferritin can be elevated or normal, due to the inflammatory response. However, since the body is in an absolute iron deficiency state in these cases, it is difficult to accurately evaluate iron homeostasis indicators (61, 62). Therefore, anemia may be an intermediary factor between AS and iron homeostasis indicators. Additionally, the anemia state caused by inflammation, complications, and treatment side effects of AS may causes changes in iron homeostasis indicators.

Considering this, inflammation or anemia may be simultaneously related to AS and the four iron homeostasis indicators in this study (ferritin, serum iron, TIBC, TSAT). AS-related anemia and inflammation-induced upregulation of hepcidin may account for the observed association between AS and the four iron homeostasis indicators. Nonetheless, this does not provide evidence of a direct genetic causal relationship between AS and iron homeostasis indicators. Furthermore, these indicators are susceptible to other diseases that cause inflammation and anemia.

This research had limitations. The data were limited to patients of European ancestry, therefore the conclusions drawn may not be applicable to other populations (63). A comprehensive and clear database is required for further research. Furthermore, there were relatively few iron homeostasis indicators in this study. Additional indicators should be selected for subsequent research to increase the robustness of the results. Finally, the exposure factors in this study were the effects of mutated genes, which may differ from the actual exposure in terms of time and dose.

## 5. Conclusion

In conclusion, our results suggest that the four iron homeostasis indicators have no clear causal relationship with AS with respect to genetic factors. Moreover, there may be a correlation between AS and iron homeostasis indicators on other levels beyond genetics. Additional mechanistic links between the two remain unclear and should be further examined in future research.

## Data availability statement

Publicly available datasets were analyzed in this study. This data can be found at: https://www.decode.com/; https://www.finngen.fi/en.

## Author contributions

PX and MY designed the study. MY, HY, KX, and JX analyzed the datasets and interpreted the results. HZ and RF downloaded the data. JW provided software support. MY wrote and edited the manuscript. PX provided the foundation and support. All authors contributed to the article and approved the submitted version.

## Funding

This work was financially supported by the National Natural Science Foundation of China (No. 82072432).

## Conflict of interest

The authors declare that the research was conducted in the absence of any commercial or financial relationships that could be construed as a potential conflict of interest.

## Publisher’s note

All claims expressed in this article are solely those of the authors and do not necessarily represent those of their affiliated organizations, or those of the publisher, the editors and the reviewers. Any product that may be evaluated in this article, or claim that may be made by its manufacturer, is not guaranteed or endorsed by the publisher.

## Supplementary material

The Supplementary material for this article can be found online at: https://www.frontiersin.org/articles/10.3389/fnut.2023.1047640/full#supplementary-material

## Footnotes

^1^ https://www.decode.com/

^2^ https://www.finngen.fi/en

^3^ https://finngen.gitbook.io/documentation/

^4^ http://www.phenoscanner.medschl.cam.ac.uk/phenoscanner

^5^ https://ldlink.nci.nih.gov/
