# Supplementary figures and images for: No evidence of a genetic causal relationship between ankylosing spondylitis and iron homeostasis: A two-sample Mendelian randomization study
Source: Front Nutr. 2023 Mar 23;10:1047640. doi: 10.3389/fnut.2023.1047640 (PMC10077893; doi:10.3389/fnut.2023.1047640)

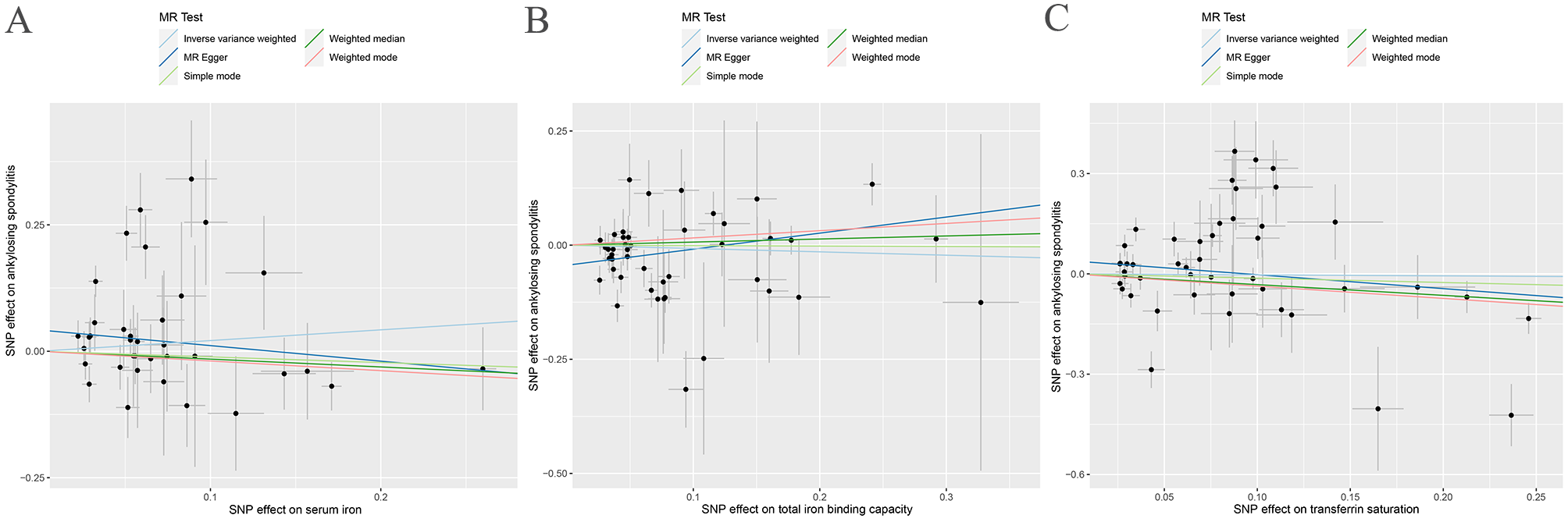

Supplement: Supplementary file 5 [file Image_1.tif]

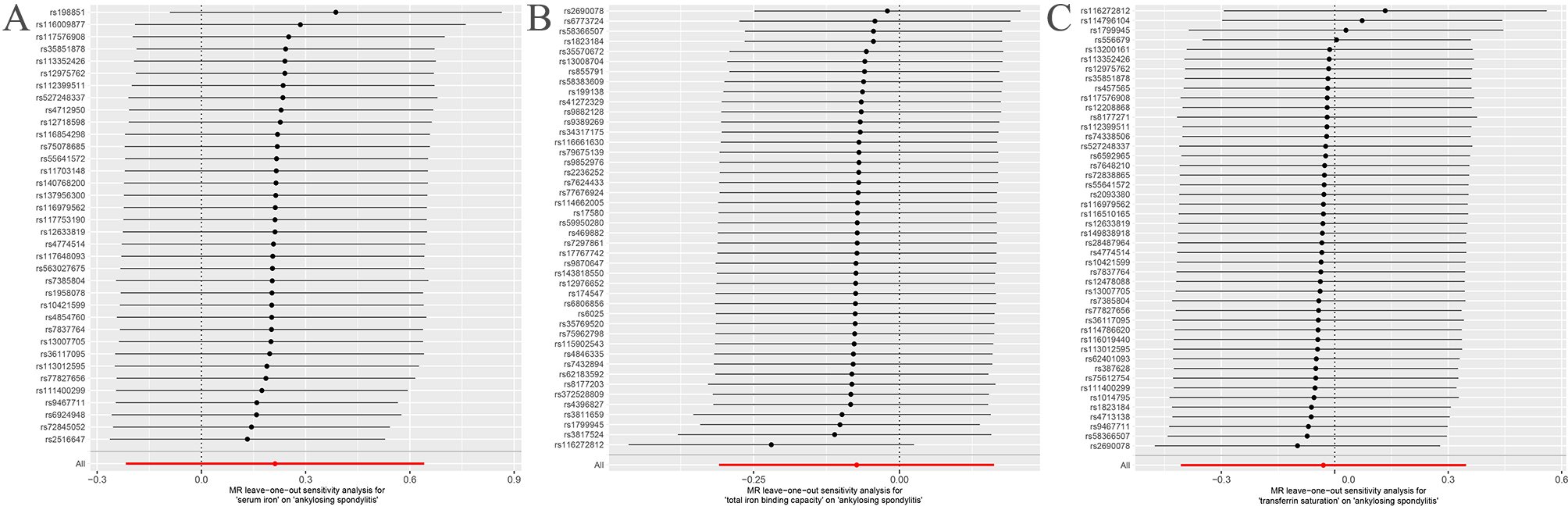

Supplement: Supplementary file 6 [file Image_2.tif]

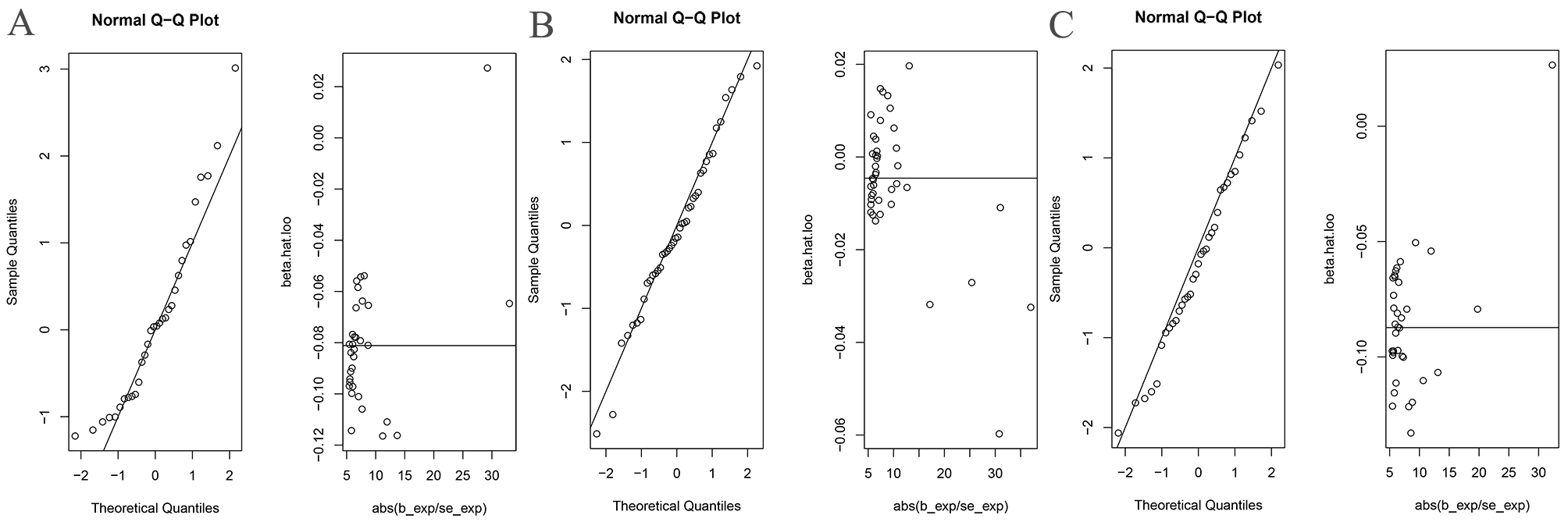

Supplement: Supplementary file 7 [file Image_3.TIF]
